# Design of Cost-Efficient Optical Fronthaul for 5G/6G Networks: An Optimization Perspective

**DOI:** 10.3390/s22239394

**Published:** 2022-12-01

**Authors:** Abdulhalim Fayad, Tibor Cinkler, Jacek Rak, Manish Jha

**Affiliations:** 1 Faculty of Electrical Engineering and Informatics, Budapest University of Technology and Economics (BME), 1117 Budapest, Hungary; 2 Faculty of Electronics, Telecommunications and Informatics, Gdańsk University of Technology, G. Narutowicza 11/12, 80-233 Gdańsk, Poland

**Keywords:** 5G, 6G, CRAN, fronthaul, TCO, cost-efficiency, ILP, genetic algorithm, K-means algorithm, heuristics

## Abstract

Currently, 5G and the forthcoming 6G mobile communication systems are the most promising cellular generations expected to beat the growing hunger for bandwidth and enable the fully connected world presented by the Internet of Everything (IoE). The cloud radio access network (CRAN) has been proposed as a promising architecture for meeting the needs and goals of 5G/6G (5G and beyond) networks. Nevertheless, the provisioning of cost-efficient connections between a large number of remote radio heads (RRHs) in the cell sites and the baseband unit (BBU) pool in the central location, known as the fronthaul, has emerged as a new challenge. Many wired and wireless solutions have been proposed to address this bottleneck. Specifically, optical technologies presented by passive optical networks (PONs) are introduced as the best suitable solution for 5G and beyond network fronthaul due to their properties of providing high capacity and low latency connections. We considered time and wavelength division multiplexed passive optical networks (TWDM-PONs) as a fronthaul for 5G and beyond. Taking that into consideration, in this paper, we propose an integer linear program (ILP) that results in the optimal optical fronthaul deployment while minimizing the total cost of 5G and beyond instances. However, for larger network instances, solving the ILP problem becomes unscalable and time-consuming. To address that, we developed two heuristic-based algorithms (the K-means clustering algorithm and the one based on the genetic algorithm—GA). We evaluated the suitability of our proposed ILP and heuristic algorithms in simulations by utilizing them to plan different network instances (dense and sparse).

## 1. Introduction

**Figure 1:**
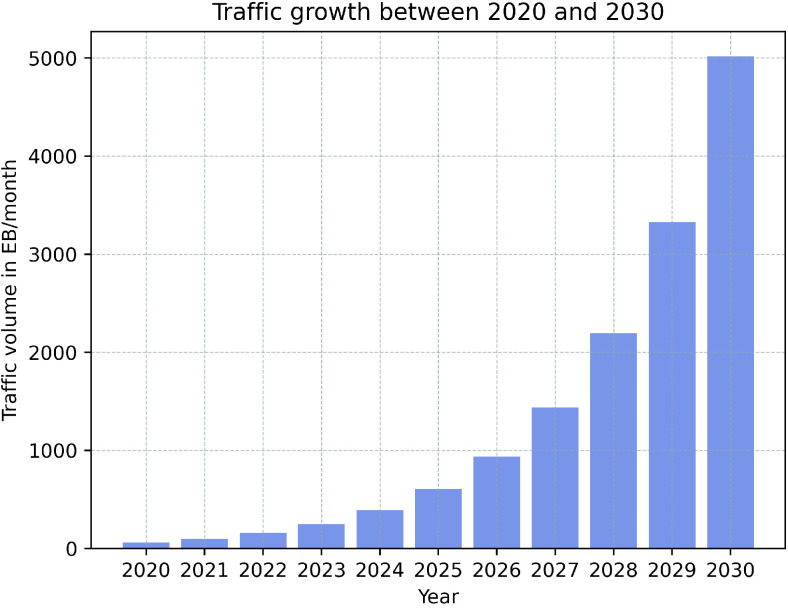
Predicted mobile data traffic per month according to the ITU-R Report M.2370-0 from 2020 to 2030.

**Figure 2:**
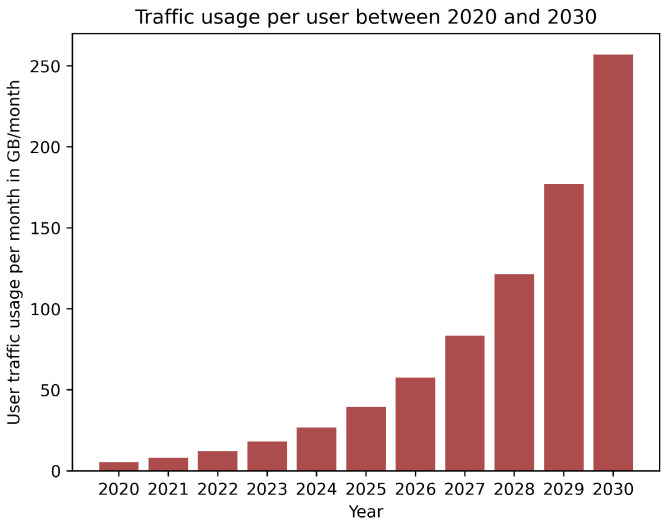
Predicted user data traffic per month according to the ITU-R Report M.2370-0 from 2020 to 2030.

**Figure 3:**
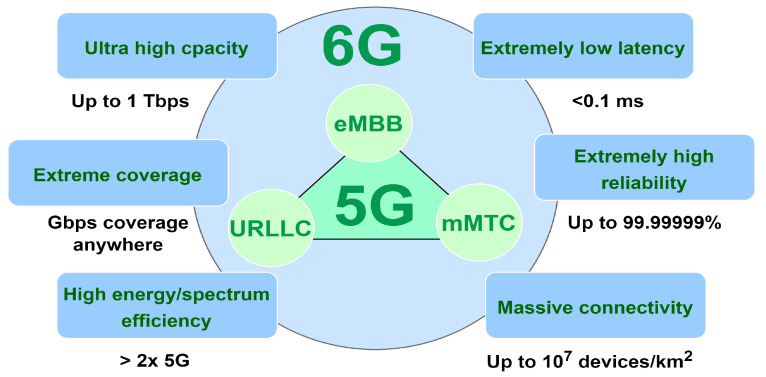
Requirements for 5G and 6G mobile technologies.

Moving toward a fully connected society, in conjunction with the rapid development of emerging applications such as the Internet of Everything (IoE), Industry 4.0, virtual reality (VR), and 8K video streaming, has resulted in unexpected traffic volume over telecommunication networks. According to the ITU-R Report M.2370-0, mobile data traffic is expected to be higher than 5036 EB per month in 2030, as shown in Figure 1, while the user mobile data usage will rise from 5 GB in 2020 to more than 250 GB per month in 2030 as in Figure 2 [1]. To handle that massive amount of traffic, industry and academia are working day and night to develop new generations of mobile communication systems. Recently, the fifth generation (5G) has been gradually deployed for commercial use across various markets [2]. The 5G network is eager to provide a fully connected society and has three main use cases: enhanced mobile broadband (eMBB), massive machine-type communications (mMTC), and ultra-reliable low-latency communications (URLLC) [2]. eMBB aims at providing high data rate communications and improving the performance of mobile networks in terms of capacity and coverage, which is crucial to provide mobile broadband connectivity to meet the massive value of data traffic. The other target is to assure a data rate of up to 20 Gbps in high-density populated areas. mMTC aims to provide the connection to a massive number of devices, such as Internet of Things (IoT) nodes, which leads to achieving the goal of a connected world. This requires shifting from serving traditional human clients in LTE systems to machine-to-machine (M2M) communications to support high bandwidth applications such as augmented/virtual reality (AR/VR) and streaming. Finally, URLLC indicates mission-critical applications (i.e., autonomous driving, vehicle-to-everything (V2X) communications, intelligent transportation systems (ITSs), and remote surgery), which require guaranteed connection and low latency. This implies that the communication link should always be available to transfer data in a short period and with high reliability [3]. However, as mobile network capacity and the number of connected devices increase, along with the introduction of new traffic patterns and service diversification, there will be a significant gap between 5G capabilities and market requirements expected after 2030. Therefore, research activities have been ongoing in order to specify requirements and identify potential radio technologies and architectures for the next big evolution in the telecommunications industry, known as 6G [3,4,5,6]. To this end, 6G is expected to provide extremely low latency connectivity, high peak data rate, high 3D coverage, significant capacity, and higher energy and spectral efficiency than 5G, as shown in Figure 3. Table 1 provides a comparison between 5G and 6G.

However, a traditional architecture for any telecommunication system consists of a number of base stations (BSs) as a medium connecting the end users to the core network. From 2G to 4G, the main deployment scenario was based on the distributed radio access network (DRAN). In DRAN, the baseband unit (BBU) and the remote radio head (RRH) were co-located at the cell site [7]. At the same time, in 5G and beyond, the deployment scenario has a centralized architecture called a cloud radio access network (CRAN). The main architecture of CRAN consists of three components: the BBU pool including a large number of BBUs with centralized processors; the RRHs with antennas located at the cell site; and the fronthaul network that connects RRHs to BBUs via high capacity and low latency connections. The design of the fronthaul has been one of the most significant challenges on the road to CRAN realization, where fully processed high-bandwidth signals must be transmitted over the fronthaul from the BBU pool to the intended RRHs. For that, it must offer low-latency and high-capacity connections in a cost-effective manner. To do that, many wired and wireless technologies can be used to realize the fronthaul, including optical fiber, microwave, millimeter waves, and free space optics. Optical fiber technologies represented by optical access networks are the best option to meet the fronthaul requirements due to their inherent characteristics, as they are capable of providing high-capacity and low-latency connections [8,9]. In this work, we focus on planning and designing an efficient optical access system for 5G and beyond fronthaul, which is a crucial and complex problem due to the high density of 5G and 6G networks, where deploying 5G and 6G optical fronthaul networks would necessitate massive investments. Therefore, an efficient and cost-effective solution for the optimal design would be required. Taking that into account, the key contributions of this work can be summarized as follows:

1. Formulation of an optimization framework based on ILP to obtain the optimal solution for 5G and beyond optical fronthaul design problems while minimizing the overall cost of the network.
2. A proposal of two sub-optimal methods based on the K-means clustering and genetic algorithms, respectively, to overcome the ILP scalability issue.
3. Assessment of the applicability of our proposed solutions by using them to optimize the deployment in two different scenarios (dense and sparse configurations).

The rest of the paper is organized as follows. Section 2 reviews the RAN architectural developments and the enabling technologies for 5G/6G fronthaul; the related works are discussed in Section 3. The problem description and the cost model are provided in Section 4. Section 5 formulates the proposed ILP model and the heuristic schemes. The case study and the obtained results are discussed in Section 6. Finally, Section 7 concludes the paper.

## 2. xRAN Architecture Developments and Fronthaul Enabling Technologies

To meet the growth of mobile traffic presented by the newly emerged applications, RANs have been undergoing several designs and technological developments. As a result, in recent years, several RAN architectures have been proposed. CRAN is a radio access network architecture based on cloud computing. In the CRAN architecture, BBUs are relocated from individual base stations to a centralized location, often called a BBU pool, while the RRHs are located on the antenna side. The BBU pool connects to the RRHs using high-bandwidth and low-latency links forming the fronthaul [7]. The heterogeneous cloud RAN (HCRAN) has been proposed due to the high need to deploy 5G dense networks. Its architecture is based on heterogeneous networks (HetNets) and cloud computing, where the macro base stations BSs provide network control, mobility management, and system performance enhancement, while micro BSs and RRHs improve the capacity of the system and lower the power of transmission [10]. The RRHs enable signal processing and radio frequency functions. In contrast, the BBU pool includes other physical baseband processing functions associated with the upper layers. With the new virtualization development, a new RAN architecture, the fog radio access network (FRAN), has been proposed. It is an architecture that makes benefits from CRAN and fog computing, aiming to overcome the exponential growth of mobile traffic and improve the quality of service (QoS) for end-users. FRAN was first proposed to fully utilize caching and AI at the network edge [11]. FRANs have several obvious advantages, including real-time signal processing and flexible networking at the network edge. FRANs adapt to the changing traffic and radio environment, relieving the fronthaul and BBU pool of capacity and transmit latency burdens.

Recently, Open RAN has been identified as a trend in mobile network architecture that typically refers to a disaggregated radio access network with open interfaces between network components sourced from multiple vendors [12]. The network functions in the Open RAN architecture are disaggregated into radio units (RU), distributed units (DU), and centralized units (CU), with open interfaces between them. Virtualization and containerization are also options for the RU, DU, and CU functions. This can lead to lower TCO and higher QoS levels. In consequence, all the designs mentioned above have the same goals: to provide high bandwidth and low latency connections to a greater number of end-users while lowering the capital expenditure (Capex) and operational expenditure (Opex). However, the main challenge for all proposed xRAN architectures is the cost of the fronthaul, where a low-latency, high-capacity fronthaul is required to transmit baseband signals between the RRHs and the BBU.

This paper considers the CRAN architecture, which can be generalized to all other architectures mentioned in this section.

### 2.1. CRAN Architecture and Functional Splits

**Figure 4:**
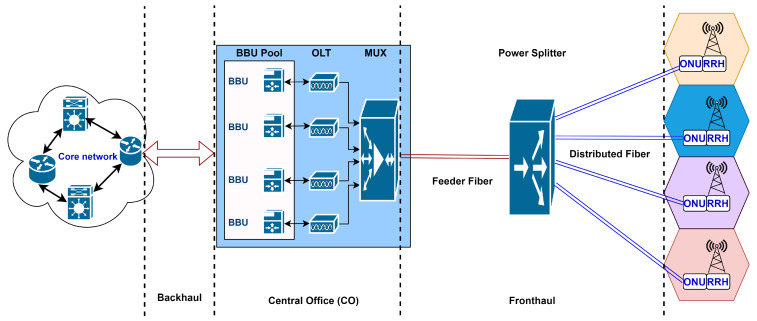
Cloud radio access network architecture.

In contrast to DRAN, the base station is divided into two parts in the CRAN architecture, as shown in Figure 4. The RRH remains at the cell site with few functional responsibilities; at the same time, the BBUs are aggregated into a centralized location. The link between the RRH and the BBU is called the fronthaul, where the most common interface/protocol used between RRH and BBU in fronthaul networks is the common public radio interface (CPRI) [13]. CRAN provides better network performance in almost every key performance indicator compared to traditional DRAN [14]. The main advantages of 5G CRAN are:

1. Cost and footprint efficiency as it requires less hardware.
2. Low energy consumption.
3. Simple and flexible architecture.
4. Resource and infrastructure sharing.
5. Increased efficiency of network upgrades and enhancements.
6. Ease of testing and maintenance.

**Figure 5:**
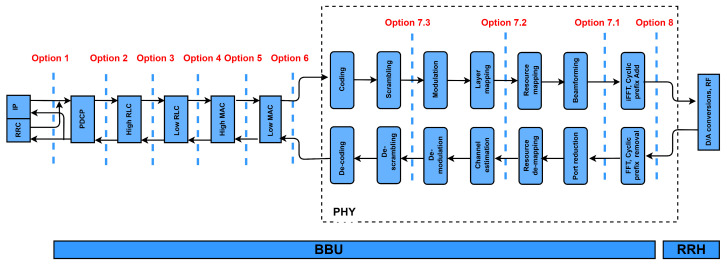
RAN functional split options.

Nevertheless, the main drawback of 5G CRAN is the cost of the fronthaul. Furthermore, the fronthaul requires high-capacity and low-latency connections. For instance, a sector configured as 64 × 64 MIMO with 20 MHz bandwidth requires about 64 Gbps for fronthaul while guaranteeing a low latency connection equal to 100 μs. For that, the 3rd generation partnership project (3GPP) has introduced several functional split options, as shown in Figure 5, which are initially identified as split points 1–8 between the BBU and the RRH in the CRAN architecture [15]. Option 8 represents a fully centralized RAN in which all baseband functions are centralized in a BBU pool, whereas option 1 denotes a traditional network architecture in which baseband functions are allocated at the cell site.

The 3GPP RAN split options are classified as high layer (options 2 and 3), medium layer (options 5 and 6), and low layer (options 7 and 8) [16,17]. The capacity requirements of each split option, presented in Table 2, are calculated using a 5G system with 200 MHz of bandwidth, 64 QAM, an 8x12 MIMO antenna array, and 96 antenna ports, as in [18].

### 2.2. Technologies Enabling 5G and beyond Fronthaul

One of the main obstacles for 5G and beyond deployment is designing a cost-effective fronthaul network to transport vast amounts of data with low latency.

This section provides a brief overview of different potential solutions for 5G CRAN fronthaul networks. Table 3 summarizes the main differences between these technologies.

**Point to point fiber optic (P2P):** P2P provides a direct, dedicated fiber link that connects the BBU and the RRH to each other on a private, high-speed fiber connection of up to 100 Gbps. This type of high-capacity connection is required for the fronthaul segment for splitting options 7.1 and 8 of 5G CRAN [19]. However, it is not a cost-effective solution as the cost of laying fiber cables reaches up to 200 USD/m and 30 USD/m in sparse and dense areas, respectively [20].

**Passive optical networks (PON):** Passive optical networks (PONs) are critical to the success of 5G and beyond networks. PON is a point-to-multipoint technology that sends data from the central office to the end user via a passive splitter, allowing for efficient use of fiber resources. Furthermore, PON has low-latency connections and can handle a large volume of data traffic [21,22]. ITU-T and full-service access network (FSAN) proposed next-generation passive optical network 2 (NG-PON2) as the next evolution of PONs to meet user expectations by increasing the transmission speed in PONs to more than 10 Gbps. The requirements of NG-PON2 have been determined in ITU-T G.989 recommendation [23]. These recommendations indicate a bandwidth capacity of up to 40 Gbps on both downlink and uplink for transmission distances up to 40 km. TWDM-PON, which combines the advantages of more wavelengths provided by WDM-PONs and a large number of users per wavelength provided by TDM-PONs, is regarded as the best option for meeting the NG-PON2 requirements. One of its primary applications is to handle mobile fronthaul streams with low latency and high capacity [24,25] and can support functional split options ranging from 1 to 6, 7.2, and 7.3 [19].

**Copper-based links (xDSL):** xDSL refers to different types of digital subscriber line (DSL) technologies [26]. In previous generations, they have been a popular solution for wireless network backhauling and were widely deployed for fixed telecommunication infrastructure. However, xDSL can not be considered a robust solution for 5G CRAN fronthaul due to bandwidth limitations.

**Free-space optics (FSO):** The use of light waves rather than radio waves to set up a link in free space is known as free-space optics (FSO). When compared to fiber-based fronthaul, the FSO, also known as fiber optics without fiber, offers a cost-effective alternative for fronthaul links, allowing for a 50% reduction in fronthaul deployment costs. FSO’s main advantages are its ability to provide low power consumption, high capacity, low price, and license-free option, and it can be used to set up point-to-multipoint links. The performance of FSO is often affected by visibility and adverse weather conditions such as fog, snowfall, or rain, making it unsuitable for long distances or non-line of sight (NLoS) links [27,28,29].

**Microwaves:** Microwave links are among the popular technologies that connect the base stations to the core network. They can be operated in the licensed spectrum from 6 GHz to 42 GHz and implemented as a point-to-point or point-to-multipoint architecture. Although the microwave links are less affected by bad weather conditions as FSO and can serve larger transmission distances, they require LoS and depend on licensed frequencies, which makes them an expensive option [30].

**Millimeter waves (mmWaves):** The mmWaves spectrum covers frequencies from 50 GHz to 300 GHz and includes the V-band (60 GHz), E-band (70/80 GHz), W-band (100 GHz), and D-band (150 GHz) [31]. mmWaves technology has gained attention as a promising solution to meet the fronthaul of 5G CRAN. It provides high capacity and low latency connection. The drawbacks are the atmospheric and rain attenuation that may become extremely high at mmWaves frequencies, making them sensitive to weather conditions [32].

As a consequence, optical technologies seem to be the best solution to handle the fronthaul data traffic for 5G and 6G networks.

## 3. Related Work

Despite the many benefits of CRAN architecture, designing a cost-effective fronthaul remains the main challenge for MNOs deploying 5G and beyond CRAN. For that reason, recently, designing a cost-effective optical fronthaul for CRAN has received significant attention from researchers. Musumeci et al. in [33] investigated a BBU placement problem in a CRAN linked by a WDM optical network. The optimization problem was formulated as an ILP with the goal of minimizing the total network cost, which was represented by either the number of active BBU sites or the number of fibers used to transport traffic in the network. Chen et al. in [34] proposed a cost-efficient DRAN architecture for deploying 5G small cells. The authors considered TWDM-PON as a backhaul connection and proposed an algorithm to find the best locations of the network equipment in order to reduce deployment costs. Klinkowski et al. [35] proposed a cost-effective ILP model for the placement of RRHs, BBUs, and optical fibers in 5G radio-optical communication networks (5G-RONs). Ranaweera et al. in [19,36] proposed an ILP-based optimization framework that jointly optimizes the wireless access and the optical transport network for 5G and 6G cellular networks. Meysam et al. in [37] proposed an ILP and a genetic algorithm to minimize the TCO of CRAN for 5G small cells and assess the cost of migrating to a CRAN-based TWDM-PON architecture. Two scenarios were considered: a complete network function centralization and a partial network function centralization via function splitting. Marotta et al. in [38] proposed an ILP model to evaluate the optimal deployment of 5G CRAN fronthaul using point-to-point optical fiber and microwave links under delay constraints in a brownfield scenario. Ranaweera et al. in [39,40] proposed an optimization framework for optimally planning a small cell network deployment simultaneously with the backhaul network while meeting the different needs of the networks making benefits from the existing fiber infrastructure. Kolydakis et al. in [41] provided a TCO comparison of wireless and fiber technologies for 5G fronthaul and backhaul solutions, resulting in the fiber architecture being more cost-effective than the wireless one in high-density areas. Jarray et al. in [42] proposed Capex and Opex cost modeling, as well as a mixed integer linear programming (MILP) optimization for large-scale mesh networks based on column generation techniques and the rounding-off heuristic. Yeganeh et al. in [43] analyzed the Capex and Opex of CRANs and mathematically formulated the cell-BBU pool assignment problem, taking into account fronthaul network expenditure. Tonini et al. [44] presented a hybrid fronthaul solution for CRAN based on optical fibers and free space optics (FSO) to improve fronthaul flexibility and reduce deployment costs. Two ILP-based design strategies are proposed for both greenfield and brownfield deployments. Arévalo et al. in [45] proposed a set of heuristic techniques to find the optimal placement of RRHs as well as the routes of the fiber-based fronthaul at the same time. The authors assessed the cost-effectiveness of using digital signal processing (DSP) assisted channel aggregation techniques in fronthaul links. In [25], we proposed an ILP formulation that optimizes the TCO for 5G using the CRAN architecture at various delay thresholds. We considered TWDM-PON as a fronthaul with different splitting ratios. Carapellese et al. in [46] investigated the best location of BBUs in a WDM aggregation network. The authors compared the impact of two different fronthaul transport options, the optical transport network (OTN) and overlay, on BBU centralization. Despite the aforementioned contributions, a bottleneck still remains in designing and deploying a cost-effective optical fronthaul for 5G and beyond networks. To the best of our knowledge, most of the literature did not provide insight study about planning a cost-effective optical fronthaul for 5G and beyond networks while considering the optimal solution for small problems along with heuristic solutions for larger problem instances.

## 4. Problem Description

In this paper, we consider a scenario in which a mobile network operator (MNO) aims to plan an optical network to provide fronthaul connectivity services to several RHHs toward a centralized BBU pool for various areas. In essence, the problem discussed in this study is to minimize the total cost of ownership (TCO) of 5G and beyond deployments both in terms of Capex and Opex while finding the optimal location for network equipment. The most important is the optimal deployment of the optical fronthaul as deploying optical fiber cables is too expensive. Any unplanned network deployment would exponentially increase the total network setup cost. To ensure that the network is deployed in such a way so that the setup cost of the network can be kept minimum and covers all the planned areas efficiently, below we discuss each optimization aspect in detail. The problem can be split into subproblems as follows:

1. **Power splitter placement:** Depending on the RRH location and splitter ratio capacity, we have to determine the best possible location to assign splitters to RRHs according to their maximum ratio capacity. The shortest distance and lowest delay are used to divide all RRHs into groups. This is a clustering problem since each set of RRHs is assigned to one splitter and is known to be NP-complete [47].
2. **BBU pool placement:** According to the splitter locations, the optimal location for the BBU pool is near the center of the splitters group in order to keep the overall length of the link between the splitters and the BBU pool within the group to a minimum.
3. **Fronthaul deployment:** To find the optimal fronthaul deployment over the network, all RRHs must be connected to the splitter based on the shortest path possible, and all splitters must be linked to the BBU pool according to the shortest distance.

Our optimization problem addresses the following questions:

1. How to establish RRH groups that are all linked to one power splitter?
2. How to find the optimal location for the BBU pool while resulting in the minimal cost?
3. How to find the shortest path from the RRH to its splitter and from the splitter to the BBU pool?

### Cost Model

This section provides a TCO cost model that considers the optical fronthaul based on TWDM-PON architecture, as well as the Capex and Opex of deploying 5G and beyond. In our study, we assume that only one operator can serve the investigated area. Furthermore, there is no infrastructure sharing, and all the infrastructure elements are associated with that operator; there is no need to lease fiber. Therefore, the TCO can be calculated as follows:

(1) $$TCO=Capex+N_{r}\cdot Opex$$

where $N_{r}$ is the number of years. (In our paper, only one year of operation is considered, which can provide the operator with the needed Opex for one year of investments. It can be predicted for several years as a linear increase.)

**Figure 6:**
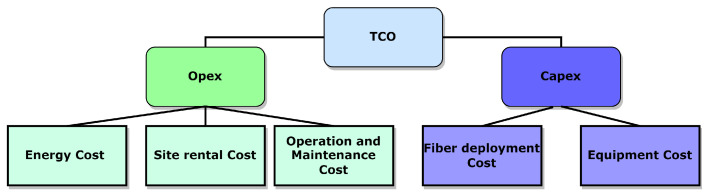
TCO breakdown.

Figure 6 depicts the model used to calculate the TCO value, as well as a breakdown of the Capex and Opex costs. This framework for cost assessment is derived from the TCO model described in [25]. The Capex and Opex will be described in detail in the following section, with the objective function to minimize the TCO of the network.

## 5. ILP Formulation and Heuristic Solutions

An integer linear program (ILP) is a popular tool for finding optimal solutions to linear problems. A proper ILP formulation can also serve as a reference. The performance of any given heuristic can be assessed by comparing it to the optimal solution obtained for the ILP.

In this section, we propose an ILP-based optimization framework with the goal of minimizing the TCO of CRAN and optical fronthaul deployment, where TWDM-PON is considered as the CRAN fronthaul as illustrated in Figure 4. Each RRH and ONU are co-located together (henceforth, we use RRH). All RRHs are assigned towards the BBU pool through two aggregation levels, at first to the power splitter (PS) and then to the arrayed wavelength grating (AWG) that is allocated with the optical line terminal (OLT) and the BBU pool. The formulation of the ILP is illustrated as follows.

*Input*: locations of all RRHs; potential locations of BBU pool; potential locations of power splitters; BBU capacity; splitting ratio; the maximum distance allowed between the BBU pool, power splitters, and RRHs; different cost values.

*Objective*: the optimal placements of BBU pools and power splitters, the minimum amount of equipment needed, and optimal fronthaul deployment.

*Output*: minimized TCO.

Below are the network data sets, parameters, decision variables, objective functions, and constraints.

### 5.1. Network Data Sets and Input Parameters

Table 4 illustrates the considered input parameters and the network data sets.

### 5.2. Decision Variables

All decision variables are clarified in Table 5.

### 5.3. Objective Function

The Objective Function are shown:

(2) $$\begin{array}{c} Minimize:\;\underset{\mathrm{BBU}\mathrm{pool}\mathrm{cost}}{\underbrace{\sum_{b=1}^{B}\left(C_{b}+EE_{cool}\right)\beta_{b}}}+\underset{\mathrm{BBUs}\mathrm{cost}}{\underbrace{\left(C_{l}+E\cdot E_{l}\right)\kappa_{l}}}+\underset{\mathrm{OLT}\mathrm{cost}}{\underbrace{\left(C_{o}+E\cdot E_{o}\right)\kappa_{o}}}+\underset{\mathrm{RRH}\mathrm{cost}}{\underbrace{\sum_{n=1}^{N}\left(C_{n}+E\cdot E_{n}\right)\eta_{n}}}+\underset{\mathrm{AWGs}\mathrm{cost}}{\underbrace{C_{a}\kappa_{a}}}+\\ \underset{\mathrm{splitters}\mathrm{cost}}{\underbrace{\sum_{p=1}^{P}C_{n}\cdot \rho_{p}}}+\underset{O\&M\mathrm{cost}}{\underbrace{C_{O\&M}}}+\underset{\mathrm{site}\mathrm{rental}\mathrm{cost}}{\underbrace{C_{Sr}}}+\underset{\mathrm{optical}\mathrm{fronthaul}\mathrm{deployment}\mathrm{cost}}{\underbrace{C_{f}\sum_{b=1}^{B}\sum_{p=1}^{P}\sum_{n=1}^{N}\left(y_{bp}\cdot D_{bp}+y_{pn}\cdot D_{pn}\right)}} \end{array}$$

### 5.4. Constraints

1. Topology constraints
  (a) The RRH can be connected to one power splitter only:
    (3) $$\sum_{p=1}^{P}y_{pn}=1\;\forall n\in N$$
  (b) The number of RRHs that are connected to one splitter can not exceed the splitting ratio:
    (4) $$\sum_{n=1}^{N}y_{pn}\leq \zeta \;\forall p\in P$$
  (c) If there is an optical link from a splitter to an RRH, it should be installed at a viable splitter location:
    (5) $$y_{pn}\leq \rho_{p}\;\forall p\in P,\forall n\in N$$
  (d) If a splitter is used at a possible site, it must be connected to at least one RRH:
    (6) $$\sum_{n=1}^{N}y_{pn}\geq \rho_{p}\;\forall p\in P$$
  (e) Each splitter can be served by only one BBU pool:
    (7) $$\sum_{b=1}^{B}y_{bp}=1\;\forall p\in P$$
  (f) The number of BBU pools can not exceed the maximum number:
    (8) $$\kappa_{b}=\sum_{p=1}^{P}y_{bp}\leq \phi \;\forall p\in P$$
  (g) If an optical path exists between a BBU pool and a splitter, the splitter must be connected to at least one RRH:
    (9) $$\sum_{b=1}^{B}y_{bp}\leq \sum_{p=1}^{P}y_{pn}\;\forall n\in N$$
  (h) The number of needed splitters should be calculated as follows:
    (10) $$\kappa_{P}=\sum_{p=1}^{P}y_{bp}\;\forall b\in B$$
  (i) The number of BBUs should be calculated as follows:
    (11) $$\kappa_{l}=\left\lceil \frac{N}{\omega }\right\rceil$$
  (j) The number of OLTs and the number of AWGs in the network should be equal to the total number of splitters:
    (12) $$\kappa_{o}=\kappa_{a}=\sum_{p}^{P}y_{bp}\;\forall b\in B$$
2. Capacity constraints
  (a) The capacity referring to the downlink transmission must be equal to or less than the maximum downlink capacity of TWDM-PON:
    (13) $$\sum_{n=1}^{N}\alpha y_{pn}\leq \xi_{D}\;\forall p\in P.$$
  (b) The capacity referring to the uplink transmission must be equal to or less than the maximum uplink capacity of TWDM-PON:
    (14) $$\sum_{n=1}^{N}\delta y_{pn}\leq \xi_{U}\;\forall p\in P.$$
3. Distance constraints
  (a) The maximum length of the distribution fiber should not surpass the maximum specific length:
    (15) $$y_{bp}d_{bp}\leq D1_{max}\;\forall b\in B,\forall p\in P$$
  (b) The distance between each RRH and its serving BBU pool must be less than the maximum distance allowed in PONs:
    (16) $$\;y_{bp}D_{bp}+y_{pn}D_{pn}\leq D_{max}\;\forall b\in B,\forall p\in P,\forall n\in N$$

### 5.5. Heuristic Approach

When dealing with larger problems, the ILP-based approach becomes unscalable and time-consuming. In this paper, for example, the ILP-based approach does not scale well when the number of RRHs exceeds 34. Thus, heuristic solutions that can handle larger problem sizes are required because real-world problems are typically larger. Although heuristic schemes do not always provide the best solutions, they scale well. In this section, the K-means clustering algorithm and the genetic algorithm (GA) are utilized to solve the planning problem of a cost-efficient optical fronthaul for 5G and beyond.

#### 5.5.1. K-means Clustering Algorithm

K-means clustering is a machine learning clustering algorithm, and it is a centroid-based algorithm where each set of points has its centroid. The main goal of this algorithm is to minimize the sum of distances between data points and their corresponding centroid. This property is used in this paper to find the best solution for our cost-effective optical fronthaul planning problem. The K-means clustering algorithm operation is divided into three steps: initialization, squared Euclidean distance, and centroid updating [48].

*Initialization:* The first step is to use the splitter ratio to determine the size of the clusters and then randomly distribute the cluster centers across all RRH locations.

*Squared Euclidean Distance:* The next stage is to allocate each RRH to the power splitter in the cluster centroid with the shortest squared Euclidean distance (SED). The Euclidean distance is represented by:

(17) $$\mathrm{SED}=\sum_{n=1}^{N}\sum_{p=1}^{P}{\left(|N_{n}-P_{p}|\right)}^{2}$$

where $|N_{n}-P_{p}|$ is the distance between the nth RRH and the pth power splitter. The same SED will be calculated between all BBU pool locations and power splitters.

*Updating centroids:* The algorithm will next recalculate new locations of power splitters (centers of the clusters). Generally, new cluster centroids (power splitter locations) are obtained by taking the mean of all cluster data points. The following equation is used to calculate the centroid (C(s)):

(18) $$\;C(s)=\sum_{k=1}^{S}\left(|N_{n}-P_{p}|\right)/N$$

From the new power splitter locations (new centroids), each RRH is reassigned to its nearest new centroid. The process is repeated until the replication shows no changes. The pseudocode of the K-means algorithm is illustrated in Algorithm 1.
**Algorithm 1** Cost-effective optical fronthaul design algorithm based on K-means clustering.**Input:***B*, *P*, *N*, $\kappa_{N}$, $C_{f}$, $C_{o}$, $C_{p}$, $C_{n}$, $C_{b}$, $C_{a}$, $C_{O\&M}$, $C_{Sr}$, *E*, $E_{l}$, $E_{o}$, $E_{n}$, $E_{\mathrm{cool}}$, $D1_{max}$, $D_{max}$, maximum number of replications**Output:** Optimal optical fronthaul deployment, optimal TCO1:Calculate the number of required splitters $\kappa_{P}$ by dividing $\kappa_{N}$ by ζ2:**while** the constraints of Equations (3)–(6), and Equations (13)–(15) not satisfied **do**3:    Choose random power splitters locations4:    **for** maximum number of replications **do**5:        **for** all $n\in \kappa_{N}$ **do**6:           **for** all p∈P **do**7:               $\sum_{n=1}^{N}\sum_{p=1}^{P}{\left(|N_{n}-P_{p}|\right)}^{2}$8:               Based on the shortest distance, find the optimal fiber deployment between each RRH and the nearest splitter (distribution fiber)9:           **end for**10:        **end for**11:    **end for**12:**end while**13:Find the optimal locations required splitters ($\kappa_{P}\in P$)14:Calculate the total cost ($TCO_{1}$)15:Choose random BBU pool location16:**for** all $\kappa_{P}\in P$ **do**17:    **for** all b∈B **do**18: 19:        $\sum_{p=1}^{P}\sum_{b=1}^{B}{\left(|P_{p}-B_{b}|\right)}^{2}$20:        **if** the constraints of Equations (7)–(10), and (13)–(16) are satisfied **then**21:           Based on the shortest distance, find the optimal fiber deployment between each *p* and the nearest BBU pool (feeder fiber)22:        **end if**23:    **end for**24:**end for**25:Find the optimal locations of the required BBU pools ($\kappa_{P}\in B$).26:According to the constraints given by Equations (11) and (12), calculate the cost of the required number of BBUs, AWGs and OLTs27:Calculate the optimized total cost (TCO2)28:Calculate the final optimized total cost (TCO = TCO1 + TCO2)

#### 5.5.2. Genetic Algorithm

**Figure 7:**
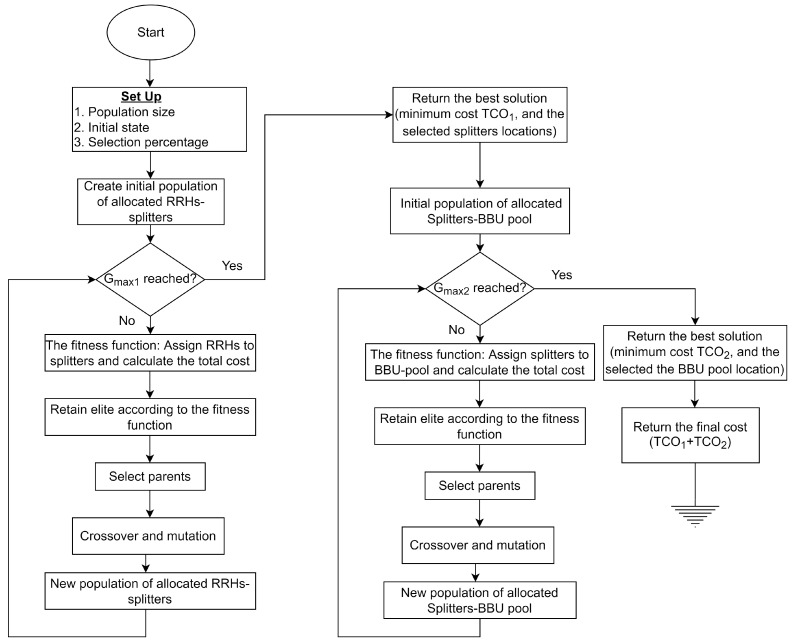
Genetic algorithm procedure flowchart.

The genetic algorithm (GA) mimics the natural selection process, in which the fittest individuals are chosen for reproduction to produce offspring for the next generation. The population “evolves” toward the optimal solution over successive generations. GA has five main stages: *Initial population*, *fitness population*, *selection*, *crossover, and mutation* [49]. The flowchart illustrated in Figure 7 explains the GA procedure from the standpoint of network design.

The following are the major steps involved in implementing the GA:

*Initial Population:* The initial population is generated randomly; the size of the population and the generation of the initial population are two significant characteristics of the population. The initial population is chosen from a large collection of solution space to reduce the computational complexity. The most common encoding method for GA is binary encoding, which is ideal for discrete solution space.

*Fitness Function:* Fitness values are generated for all chromosomes based on the problem objective function. Every time a new population is formed, the fitness function is employed to calculate the fitness values.

*Selection Process:* The next step is to follow the selection process after computing the fitness values of all starting chromosomes. The process of selecting chromosomes from a population to execute crossover and mutation operations is known as selection. The greater the fitness score, the more likely chromosomes will be chosen for further procedures. Variations should be balanced in the selection process. Some frequent techniques for picking the chromosomes for the matching pool include roulette wheel selection, tournament selection, and rank selection [49]. The circular wheel is split into $P_{pop}$ slots in the roulette wheel technique, where $P_{pop}$ represents the number of chromosomes. As a result, each chromosome has a proportional share of the wheel based on its fitness value. By hosting a tournament competition among individuals, the tournament selection technique creates selective pressure and picks the best individuals for continued operation. The rank selection approach employs a ranking mechanism based on fitness value. The worst fitness gets the highest ranking, while excellent fitness has the lowest.

*Crossover:* Offspring are generated by the crossover using a mechanism that inherits valid characters from two parents chosen from the population and can move to new regions of the search space to explore new solutions. Crossover procedures include single-point crossover, two-point crossover, multi-point crossover, and uniform crossover.

*Mutation:* After the crossover operation, the mutation is the next stage in introducing variety to the algorithm. It is essentially a procedure that randomly modifies individuals’ genes and is used to avoid the local optimum solutions. A few ways for mutation function include bit flop mutation, swap mutation, and scramble mutation. The mutation function introduces unpredictability to the chromosomes based on likelihood. The value of mutation probability should ideally be maintained low. Otherwise, the GA will revert to a random search. The pseudocode of the GA algorithm is illustrated in Algorithm 2.
**Algorithm 2** Cost-effective optical fronthaul design based on the GA.**Input:***B*, *P*, *N*,$\kappa_{n}$, $C_{f}$, $C_{o}$, $C_{p}$, $C_{p}$, $C_{b}$, $C_{a}$, $C_{O\&M}$, $C_{Sr}$, *E*, $E_{l}$, $E_{o}$, $E_{n}$, $E_{\mathrm{cool}}$, maximum number of replications, population size ($P_{pop}$), maximum number of generations ($G_{max}$), number of elites in each generation ($G_{E}$), mutation rate ($M_{mut}$), $D_{pn}$, $D_{bp}$, $D1_{max}$,$D_{max}$.**Output:** Optimal optical fronthaul deployment, optimal TCO.
1:Calculate the number of required splitters $\kappa_{P}$ by dividing $\kappa_{N}$ by ζ2:**while** Maximum number of replications not reached **do**3:    Start GA to find the optimal power splitter locations:4:    Make initial Population size ($P_{pop}$)5:    **for** all $n\in \kappa_{N}$ **do**6:        **for** all p∈P **do**7:           Assign each RRH with the power splitter in terms of the minimum distance8:           Calculate the fitness of the map and the total distance9:           Return the sorted list of fitness10:           Determine the best chromosome index and distance11:           Record initial population data12:           **for** $G\in G_{max}$ **do**13:               Get the $G_{E}$14:               Create next generation15:               Rank the routes16:               Use selection to get the next-generation parents17:               Generate matching pool18:               Produce the offspring from selected parents19:               Apply mutation20:               Return the new generation population21:               Keep the $G_{E}$ in next generation22:               Apply best chromosome search23:               Store result of each generation24:               Return result of all generations25:               Apply the cost parameters and calculate the optimized total cost (TCO1).26:           **end for**27:        **end for**28:    **end for**29:    Start GA for P and B mapping:30:    Make initial Population size ($P_{p}$)31:    **for** all $\kappa_{P}\in P$ **do**32:        **for** all b∈B **do**33:           Assign each power spitter with the BBU pool in terms of the minimum distance34:           Repeat steps (8) to (24)35:           Follow the constraints given by Eq. (10), and (11), and calculate the cost of the required number of BBUs, AWGs and OLTs.36:           Apply the cost parameters and calculate the optimized total cost (TCO2)37:        **end for**38:    **end for**39:**end while**40:Calculate the final optimized total cost (TCO = TCO1 + TCO2)

## 6. Case Study and Numerical Results

In this section, we use our proposed ILP model, K-means, and GA solutions to design the optical fronthaul of 5G and beyond networks in a cost-effective manner to demonstrate their applicability. We consider two deployment scenarios: one with a size of 5 × 5 km${}^{2}$ (dense scenario), and one with a size of 20 × 20 km${}^{2}$ (sparse scenario).

### 6.1. Simulation Setup

Simulations were run to evaluate the performance of our solutions. To solve the proposed ILP presented in Section 7, we used the commercially available CPLEX solver [50] and Python programming for K-means and GA algorithms. All simulations were conducted using a machine with Intel(R) Core(TM) i5-1035G1 CPU @ 1.00 GHz and 8 GB RAM. With that in mind, for solving the ILP the number of RRHs was 34, which was the maximum number the ILP could scale with. Then we evaluated the performance of our heuristic algorithms we considered various numbers of RRHs, i.e. 50, 100, 150, and 200. The capacity that the RRH can support was assumed to be 2.5 Gbps. We considered three different splitting rations (1:4, 1:8, and 1:16), and the TWDM-PON had a capacity of 40 Gbps for both the downlink and the uplink (symmetrical architecture). Although the proposed solutions can provide the optimal number of needed BBU pools, in this paper, we assumed that only one BBU pool location is allowed to be built. When evaluating the performance of our solutions, we considered various parameter values, which are shown in Table 6. These values are chosen from multiple studies [25,27,37,51]. However, the aforementioned parameters can be easily changed based on the data set provided by the operator.

### 6.2. Results and Discussion

**Figure 8:**
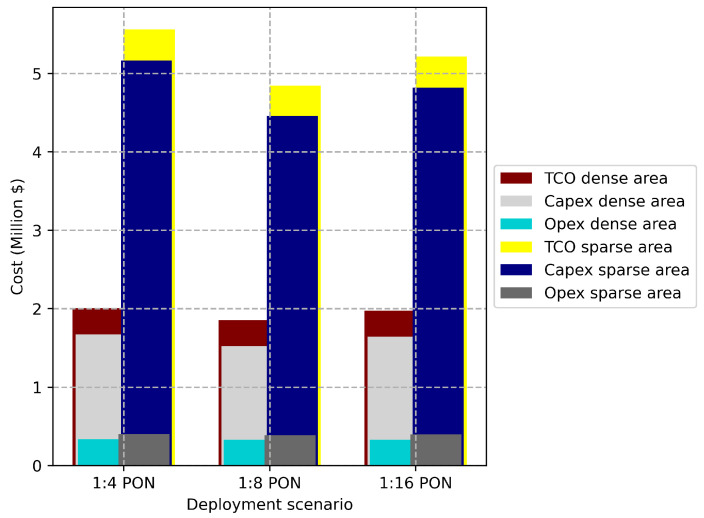
Optimal cost vs. deployment scenario for sparse and dense areas.

**Figure 9:**
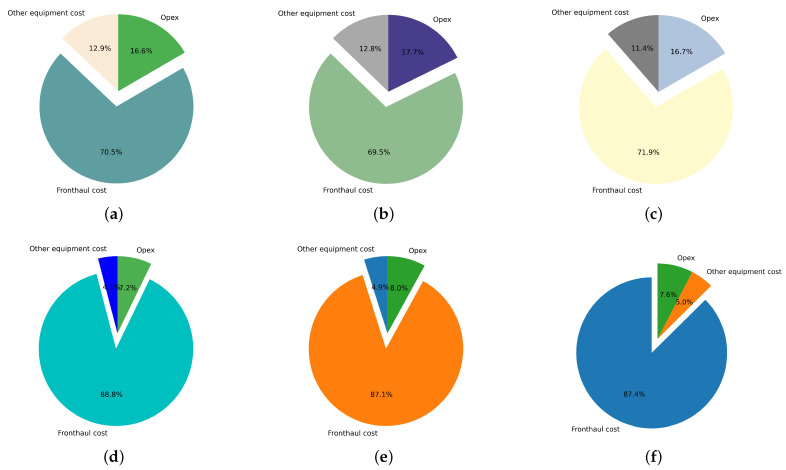
Total cost breakdown for sparse and dense areas for (**a**) 1:4 PON dense area, (**b**) 1:4 PON dense area, (**c**) 1:8 PON dense area, (**d**) 1:8 PON sparse area, (**e**) 1:16 PON sparse area, and (**f**) 1:16 PON sparse area.

Figure 8 shows the breakdown of the optimal total cost of the network in the sparse and dense deployment areas considering PON as a fronthaul with different splitting ratios (1:4, 1:8, and 1:16). We can observe that using 1:8 PON results in the most cost-efficient architecture. In the sparse area, using 1:8 PON can save up to 13% and 7.13% compared to 1:4 PON and 1:16 PON, respectively, while in the dense area, it can save up to 8% and 6.6% compared to 1:4 PON and 1:16 PON. In a sparse deployment scenario, the Capex reaches up to 93% of TCO, while in a dense scenario, up to 83.3% of the TCO. The reason is that the distances in the sparse area are larger than those in the dense area, which explains the higher costs in order to deploy longer distances of fiber cables. Figure 9 illustrates the optimal cost breakdowns in the dense and sparse deployment scenarios with different PONs. It is clear that the cost of the fronthaul is the most dominant one, due to the high investments needed for fiber deployment. Detailed cost values of Figure 9 are clarified in Table 7.

**Figure 10:**
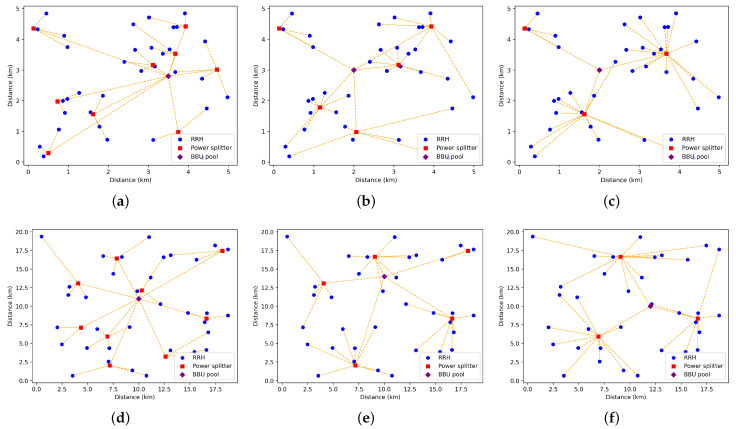
Optimal network deployment for studied areas for (**a**) 1:4 PON dense area, (**b**) 1:8 PON dense area, (**c**) 1:16 PON dense area, (**d**) 1:4 PON sparse area, (**e**) 1:18 PON sparse area, and (**f**) 1:16 PON sparse area.

Figure 10 exemplifies optimal fronthaul deployments provided by our ILP for the tested areas, considering TWDM-PON with different splitting ratios 1:4, 1:8, and 1:16 as a fronthaul. The blue dots represent RRH locations, the red squares represent splitter locations, and the purple lozenge represents the BBU pool location. Orange dashed lines represent optical fiber connections.

**Figure 11:**
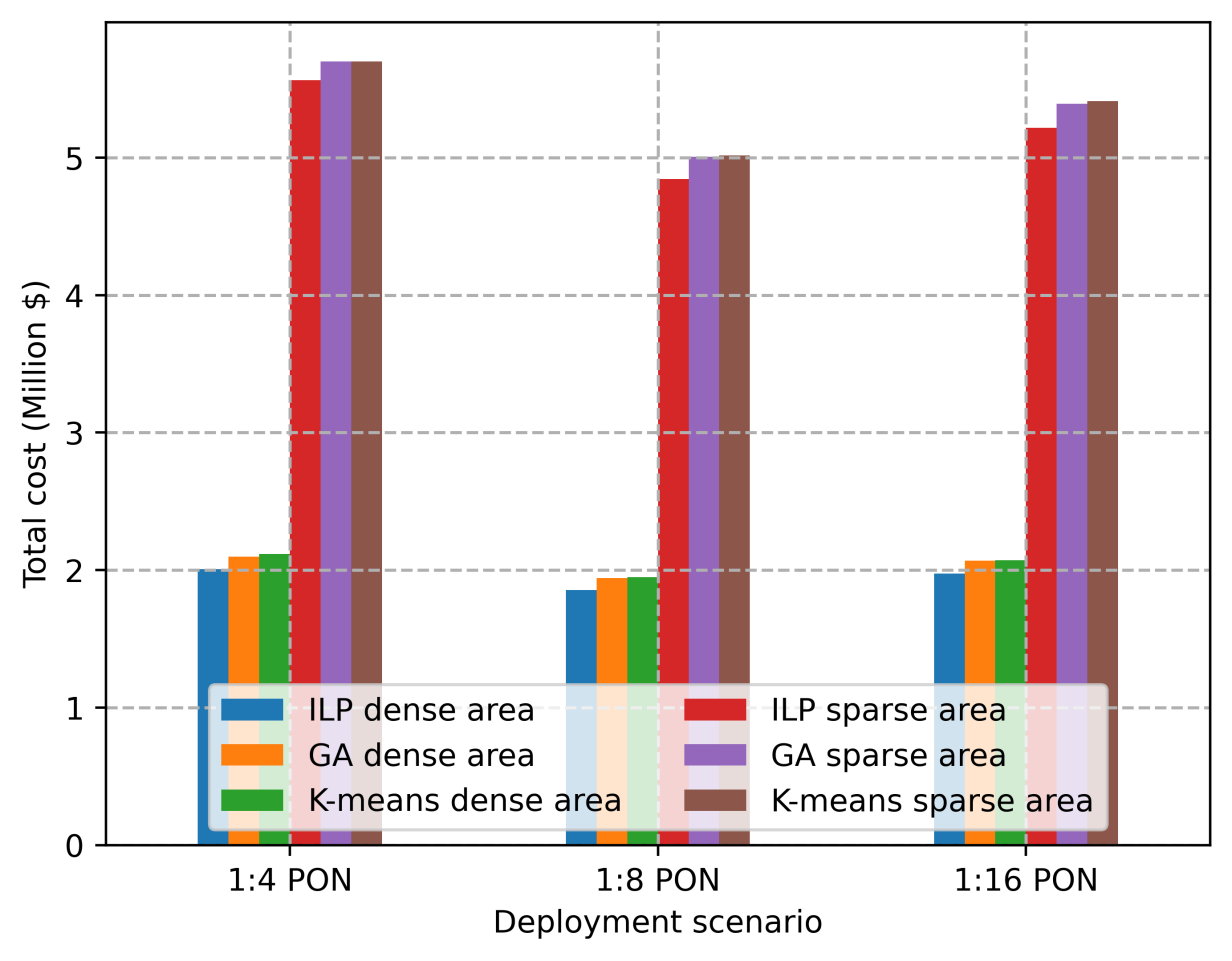
Total cost vs. deployment scenario.

**Figure 12:**
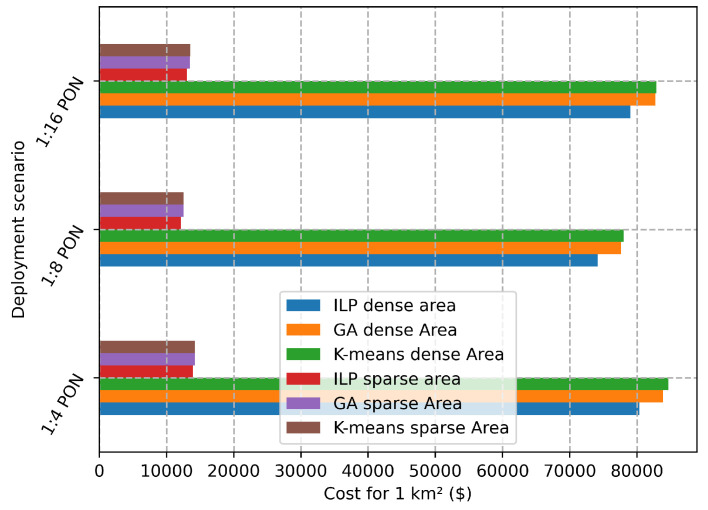
Total cost vs. deployment scenario for deploying 1 km${}^{2}$.

Figure 11 shows the comparison of the performance of the proposed K-means clustering and GA with the optimal cost provided by the ILP solution for the sparse and the dense deployment areas. We can observe that the K-means and GA perform similarly to the ILP for the number of RRHs equal to 34, which is the maximum number that the proposed ILP scales with. GA provides performance approximation 3.8% compared to the ILP, while K-means offers 4.2% approximation for the optimal solution. Figure 12 shows the exact needed costs for deploying a square area with size 1 km${}^{2}$ either in the sparse or the dense deployment. We provide these values as cost units to help MNOs to calculate the approximation costs needed for deploying larger areas.

**Figure 13:**
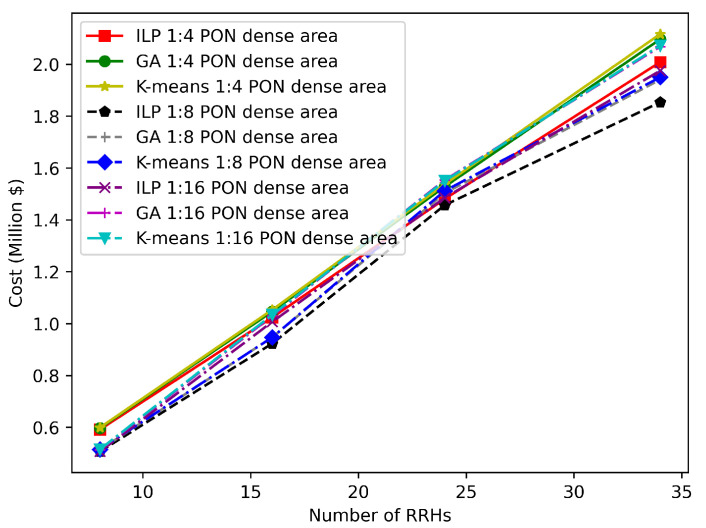
Comparison of GA and K-means solutions with the ILP solution in a dense area scenario.

**Figure 14:**
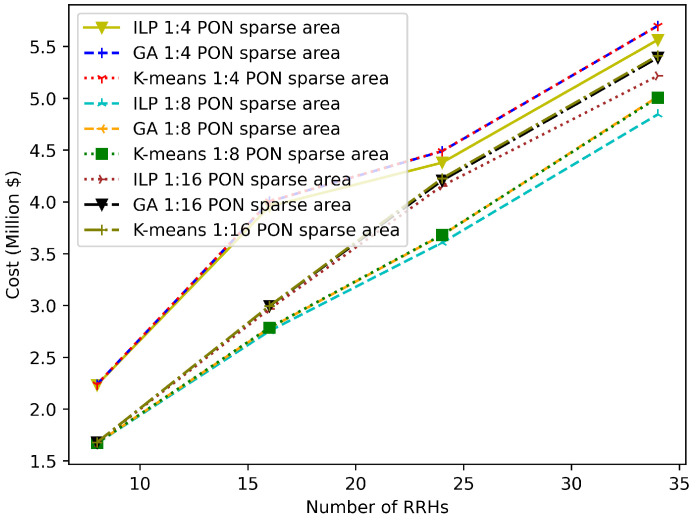
Comparison of GA and K-means solutions with the ILP solution in a sparse area scenario.

In Figure 13 and Figure 14, the optimality gap between the K-means clustering solutions, GA solutions, and the optimal solution obtained from the IBM ILOG CPLEX is shown. Moreover, the results in Figure 13 are obtained for the dense deployment area with different network sizes (8, 16, 24, and 34) considering PONs with different splitting ratios (1:4, 1:18, and 1:16), while the results in Figure 14 are presented for the sparse deployment area. It can be observed that the K-means and GA solutions coincide with the optimal solution for small network sizes. However, the optimality gap grows as the network size increases. This occurs because as the network size grows, so does the search space size, making it more difficult for the K-means and the GA to find the optimal solution.

**Figure 15:**
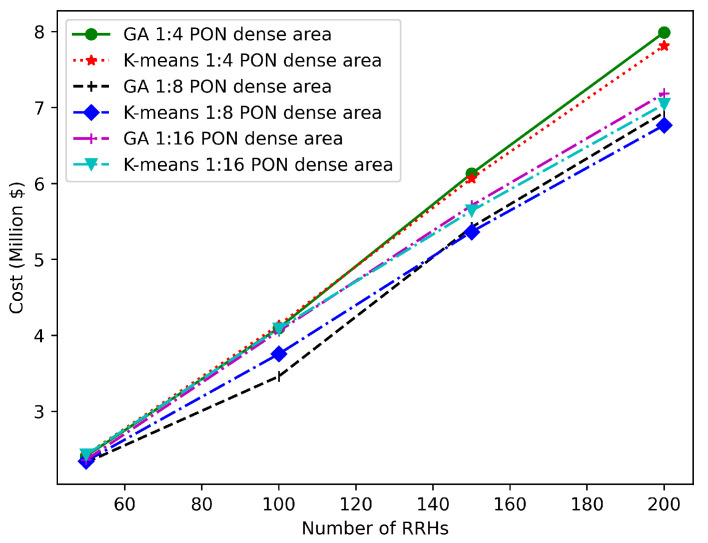
Variation in TCO for various numbers of RRHs in a dense area scenario.

**Figure 16:**
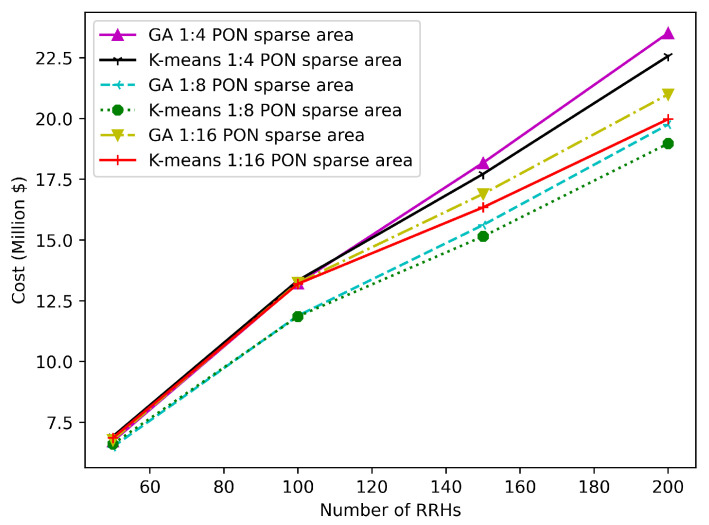
Variation in TCO for various numbers of RRHs in a sparse area scenario.

Figure 15 and Figure 16 illustrate the TCO that is needed for the sparse and dense areas, respectively, when the number of RRHs varies from 50 to 200 considering PON with different splitting ratios (1:4, 1:8, 1:16). All results in these figures are obtained by applying K-means and GA, where our ILP does not scale when the number of RRHs is higher than 34. We can observe that the GA is characterized by a better performance than the K-means for a number of RRHs less than 140, while K-means provides better results for a higher number of RRHs compared to GA (2.25% and 4.52% better performance for 200 RRHs in the dense and sparse areas, respectively).

## 7. Conclusions

The cost of optical fronthaul is one of the main challenges to be faced when deploying 5G and beyond networks. This paper investigates planning a cost-effective optical fronthaul for 5G and beyond mobile networks considering TWDM-PON with three different splitting ratios (1:4, 1:8, and 1:16). In order to achieve this, we proposed an ILP-based mathematical model that ensures the optimal solution. The objective of the optimization problem was to minimize the TCO. To do so, our proposed ILP model provided the optimal location of power splitters, the optimal location of the BBU pool, the minimum number of needed equipment, and the optimal fronthaul deployment. We used ILOG CPLEX to solve the ILP problem optimally. Despite the fact that the ILP guarantees the optimal solution, it becomes unscalable and time-consuming when solving larger-size problems. For that, we developed heuristic solutions, i.e., K-means clustering and GA-based. We demonstrated the applicability of our proposed solutions by using them to plan the optical fronthaul for 5G and beyond in two deployment areas (sparse and dense). Then, we used the proposed heuristic algorithms to solve larger-size problems with a larger number of RRHs. Moreover, our solutions can provide an acceptable cost when designing the optical fronthaul for 5G and beyond for a given territory and a set of service requirements. Another application for our solutions is as a tool for total cost estimation, which can assist mobile network operators in effectively planning their networks. As a future direction, other technologies such as microwave and free space optics can be considered as they are good alternatives for fiber optics. Additionally, other possible extensions can be focused on the adaptation of our solutions to meet the future O-RAN design by dividing the BBU into two parts (DU and CU). In this case, designing the midhaul between the CU and DU, as well as the fronthaul between the DU and the radio unit (RU) would have to be considered. Furthermore, jointly optimizing the radio and optical transport of 5G/6G networks is an important issue, where the allocation of the RU or the RRH should be included in the objective function. The RU allocation problem can be seen as a type of set-covering problem. Finally, in order to meet the openness in the future O-RAN architecture, further work should also address wavelength and bandwidth assignment techniques, which could allow more than one operator to use the same network. 

## Figures and Tables

**Table 1:** Comparison of 5G and 6G specifications.

Aspect	5G	6G
Year	2020	2030
Peak data rate (per device)	10 Gbps	1 Tbps
Maximum frequency	300 GHz	10 THz
Downlink data rate	20 Gbps	1 Tbps
Uplink data rate	10 Gbps	1 Tbps
Latency	1 ms	100 μs
Jitter	not specified	1 μs
Mobility	500 km/h	1000 km/h
Maximum bandwidth	1 GHz	100 GHz
Density of devices	$10^{6}$ devices/km${}^{2}$	$10^{7}$ devices/km${}^{2}$
Area traffic capacity	10 Mb/s/m${}^{2}$	1 Gb/s/m${}^{2}$
Peak spectral efficiency	30 b/s/Hz	100 b/s/Hz
Reliability	>99.999%	>99.9999%

**Table 2:** Fronthaul capacity requirements of various split options based on [18].

	Average Required Capacity (Gbps)									
**Split option**	1	2	3	4	5	6	7.3	7.2	7.1	8
Downlink	1	1	1	1	1	1.2	2	6	323	885
Uplink	1	1	1	1	1	1.2	3.2	2	323	885

**Table 3:** Potential fronthaul technologies.

Fronthaul Technology	Throughput	Latency	Cost	Distance	Topology
P2P Fiber	1000 Gbps	Very low	High	100 km	P2P
PON	40 Gbps	Very low	Low	40 km	P2mP
xDSL	100 Mbps	Very High	Very low	500 m	P2P
FSO	10 Gbps	Very Low	Low	5 km	P2P, P2mP
Microwave	1 Gbps	Moderate	Moderate	10 km	P2P, P2mP
mmWaves	10 Gbps	Moderate	High	1 km	P2P, P2mP

**Table 4:** Network data sets and parameters.

Notation	Description
*B*	A set of potential locations where the BBU pool, the OLT, and the AWG can be located
*P*	A set of candidate locations for splitters
*N*	A set of RRH locations
*L*	A set of BBUs
*O*	A set of OLTs
$\kappa_{b}$	Number of locations available for BBU pool placement
$\kappa_{l}$	Number of BBUs
$\kappa_{o}$	Number of OLTs
$\kappa_{a}$	Number of AWGs
$\kappa_{p}$	Number of splitters
$\kappa_{n}$	Number of RRHs, where $\kappa_{N}$=|N|
ϕ	The maximum number of BBU pools
ω	The maximum number of RRHs that can be served by one BBU
$D_{bp}$	The distance between the $b^{th}$ BBU pool and the $p^{th}$ power splitter (the feeder fiber)
$D_{pn}$	The distance between the $p^{th}$ power splitter and the $n^{th}$ RRH (the distributed fiber)
$D1_{\max}$	The maximum allowed distance for the distributed fiber
$D_{\max}$	The maximum allowed distance for the feeder fiber
ζ	Number of splits for the power splitter (splitting ratio)
$C_{f}$	The cost of fiber optic cable per meter (material and deployment cost)
$C_{o}$	The cost of the OLT
$C_{p}$	The power splitter cost
$C_{l}$	The BBU cost
$C_{n}$	The RRH cost
$C_{b}$	The BBU pool cost
$C_{a}$	The cost of AWG
$C_{O\&M}$	Operations and maintenance cost
$C_{Sr}$	Site rental cost
*E*	Energy consumption cost
$E_{l}$	Energy consumption of the BBU
$E_{o}$	Energy consumption of the OLT
$E_{n}$	Energy consumption of the RRH
$E_{cool}$	Energy consumption of the cooling system
$\xi_{D}$	TWDM-OPON downlink capacity
$\xi_{U}$	TWDM-PON uplink capacity
α	The capacity required by each RRH for downlink
δ	The capacity required by each RRH for uplink

**Table 5:** Decision variables.

Variable	Description
$\beta_{b}$	Equals 1 if the *b*th BBU pool is used; 0 otherwise
$\eta_{n}$	Equals 1 if the *n*th RRH is active; 0 otherwise.
$\rho_{p}$	Equals 1 if the *p*th power splitter is active; 0 otherwise.
$y_{bp}$	Equals 1 f the *b*th BBU pool and the *p*th power splitter are connected; 0 otherwise
$y_{pn}$	Equals 1 if the *p*th power splitter and the *n*th RRH are connected; 0 otherwise.

**Table 6:** Values of parameters.

Parameter	Value
$C_{f}$	USD 20 per m (USD 4 for purchase and 16 for trenching) [25]
$C_{o}$	2500$\sqrt{\lambda }$, where λ is the number of wavelengths (here 4)
$C_{b}$	USD 75000 [25]
$C_{l}$	USD 3600 [27]
$C_{n}$	USD 3500 [25]
$C_{a}$	$500+70\log_{2}\left(\mathrm{number}\;\mathrm{of}\;\mathrm{output}\;\mathrm{ports}\right)$ [51]
$C_{p}$	USD (30, 50, 100) for (1:4, 1:8, 1:16) splitting ratios, respectively [25]
$C_{O\&M}$	10% of equipment cost [37]
$C_{Sr}$	USD 8000 per year per RRH [25]
*E*	USD 0.15 per Watt
$E_{l}$	100 W [25]
$E_{o}$	155 W [25]
$E_{n}$	104 W [25]
$E_{\mathrm{cool}}$	500 W [25]
ω	10 RRHs
Mutation probability	0.05
Crossover probability	0.8
Number of replications	100

**Table 7:** Optimal cost breakdown for sparse and dense deployment scenarios.

Deployment Area	Dense			Sparse		
**PON architecture**	**1:4**	**1:8**	**1:16**	**1:4**	**1:8**	**1:16**
**Fronthaul cost $\times 10^{5}$ (USD)**	14.15	12.88	14.12	42.39	42.21	45.60
**Opex $\times 10^{5}$ (USD)**	3.33	3.29	3.28	3.99	3.96	3.86
**Other equipment cost $\times 10^{5}$ (USD)**	2.59	2.37	2.25	2.59	2.37	2.25

## Data Availability

Not applicable.
